# Supplementary material for: Motor crosslinking augments elasticity in active nematics
Source: Soft Matter. 2024 Feb 2;20(11):2480–90. doi: 10.1039/d3sm01176c (PMC10933839; doi:10.1039/d3sm01176c)
Supplement: SM-020-D3SM01176C-s001 [file SM-020-D3SM01176C-s001.pdf]

Electronic Supplementary Material (ESI) for Soft Matter.
This journal is © The Royal Society of Chemistry 2024

Motor crosslinking augments elasticity in active nematics

Steven A. Redford[1,2], Jonathan Colen[3], Jordan L. Shivers[4,5], Sasha Zemsky[6], Mehdi Molaei[7], Carlos Floyd[4], Paul V. Ruijgrok[6], Vincenzo Vitelli[3,4], Zev Bryant[6,8], Aaron R. Dinner[2,4,5,*], Margaret L. Gardel[2,3,4,7,*]

[1]The Graduate Program in Biophysical Sciences, University of Chicago, Chicago, IL 60637, U.S.A.
[2]Institute for Biophysical Dynamics, University of Chicago, Chicago, IL 60637, U.S.A.
[3]Department of Physics, University of Chicago, Chicago, IL 60637, U.S.A.
[4]James Franck Institute, University of Chicago, Chicago, IL 60637, U.S.A.
[5]Department of Chemistry, University of Chicago, Chicago, IL 60637, U.S.A.
[6]Department of Bioengineering, Stanford University, Stanford, CA, 94305, U.S.A.
[7]Pritzker School of Molecular Engineering, The University of Chicago, Chicago, IL, 60637, U.S.A.
[8]Department of Structural Biology, Stanford University School of Medicine, Stanford, CA, 94305, U.S.A.
[*]Correspondence: dinner@uchicago.edu, gardel@uchicago.edu

SUPPLEMENTARY TEXT

In the main text, we estimated the characteristic velocity v and characteristic length ℓ using quantities (ε and $P_{c\ell}$) derived from the kinetic simulations. Here, we consider an alternative model in which the motor velocity v_m and crosslinking probability P_{cl} are assumed to follow simple Michaelis-Menten-like dependences on [ATP]. Following [1], we assume that the motor velocity v_m takes the form

$$v_m = \left(\frac{[\text{ATP}]}{C_1 + [\text{ATP}]}\right) v_{m\infty}, \tag{S1}$$

in which $v_{m\infty}$ is the maximum velocity (corresponding to $[\text{ATP}] \to \infty$, which we take to be 1 for simplicity) and C_1 is a constant with units of concentration that represents the value of [ATP] at which $v_m = v_{m\infty}/2$. We then assume that the probability of motor attachment P_{cl} takes the form

$$P_{cl} = \left(\frac{C_2}{C_2 + [\text{ATP}]}\right) P_{cl0}, \tag{S2}$$

in which P_{cl0} is the maximum motor attachment probability (corresponding to $[\text{ATP}] = 0$, which we also take to be 1) and C_2 is a constant with units of concentration that represents the value of [ATP] at which $P_{cl} = P_{cl0}/2$. The experimental observations and simulations both suggest that $C_1 < C_2$. In Fig. S6A,B, we plot the normalized motor velocity $v_m/v_{m\infty}$ and normalized motor crosslinking probability P_{cl}/P_{cl0} for $C_1 = 80$ μM and $C_2 = 400$ μM.

The extension rate ε is assumed to be proportional to the product of the motor speed v_m and the motor attachment probability P_{cl}:

$$\varepsilon \sim v_m P_{cl}. \tag{S3}$$

As in the main text, we assume that $\alpha \sim \varepsilon^\beta$ and $K = K_0 + \kappa c_e$, where $c_e = c_p + c_m P_{cl}$, and K_0, κ, and c_m are positive constants. In the absence of passive crosslinker ($c_p = 0$), we can estimate the characteristic active length scale discussed in the main text as

$$\ell \sim \sqrt{\frac{K}{\alpha}} \sim \left(K_0 + \kappa c_m P_{cl}\right)^{1/2} \left(v_m P_{cl}\right)^{-\beta/2} \tag{S4}$$

and the characteristic velocity scale as

$$v \sim \frac{\sqrt{K\alpha}}{\eta} \sim \frac{1}{\eta} \left(K_0 + \kappa c_m P_{cl}\right)^{1/2} \left(v_m P_{cl}\right)^{\beta/2}. \tag{S5}$$

For nonzero K_0, the limiting behavior in the small concentration ($[\text{ATP}] \to 0$) limit is

$$\ell \sim [\text{ATP}]^{-\beta/2} \text{ and } v \sim [\text{ATP}]^{\beta/2} \tag{S6}$$

and the limiting behavior in the large concentration ([ATP] $\to \infty$) limit is

$$\ell \sim [\mathrm{ATP}]^{\beta/2} \quad \text{and} \quad v \sim [\mathrm{ATP}]^{-\beta/2}. \tag{S7}$$

In Fig. S6C,D, we plot the characteristic velocity v and length ℓ as a function of [ATP] for various values of the exponent β and other parameters as given in the caption. We find that, over the relevant range of [ATP], the characteristic velocity v and length ℓ exhibit the same trends observed in the experiments and kinetic simulations: a peak in v with decreasing ℓ. Note that, according to (S7), for very large concentrations ([ATP] $\gg C_2$), ℓ eventually increases with [ATP]. However, this increase is negligible over the range of [ATP] shown in Fig. S6D. For larger values of β, the variation in v and ℓ with [ATP] becomes stronger. These observations reinforce the physical interpretation in the main text that the trends in v and ℓ observed in the experiments reflect the influence of [ATP] on both nematic activity and elasticity.

REFERENCES

[1] Linnea M. Lemma, Stephen J. DeCamp, Zhihong You, Luca Giomi, and Zvonimir Dogic. Statistical properties of autonomous flows in 2D active nematics. *Soft Matter*, 15(15):3264–3272, 2019.

[2] Mehdi Molaei, Steven A. Redford, Wen-hung Chou, Danielle Scheff, Juan J. de Pablo, Patrick W. Oakes, and Margaret L. Gardel. Measuring response functions of active materials from data, March 2023. arXiv:2303.17785 [cond-mat, q-bio].

LEGENDS FOR SUPPLEMENTARY MOVIES

Movie S1 Nematics driven by tetrameric motors at varying concentrations of ATP. (A) 6 μM, (B) 16 μM, (C) 40 μM, and (D) 100 μM ATP. Note the polarization effect which renders filaments oriented along the Y axis of the movie brighter than those oriented orthogonally. All movies or collected on the same day from the same protein stocks.

Movie S2 Nematics driven by trimeric motors at varying concentrations of ATP. (A) 6 μM, (B) 16 μM, (C) 40 μM, and (D) 100 μM ATP. All movies or collected on the same day from the same protein stocks.

Movie S3 Nematics driven by octameric motors at varying concentrations of ATP. (A) 50 μM, (B) 100 μM, (C) 250 μM, (D) 500 μM, (E) 750 μM, and (F) 1000 μM ATP. All movies or collected on the same day from the same protein stocks.

SUPPLEMENTAL FIGURES

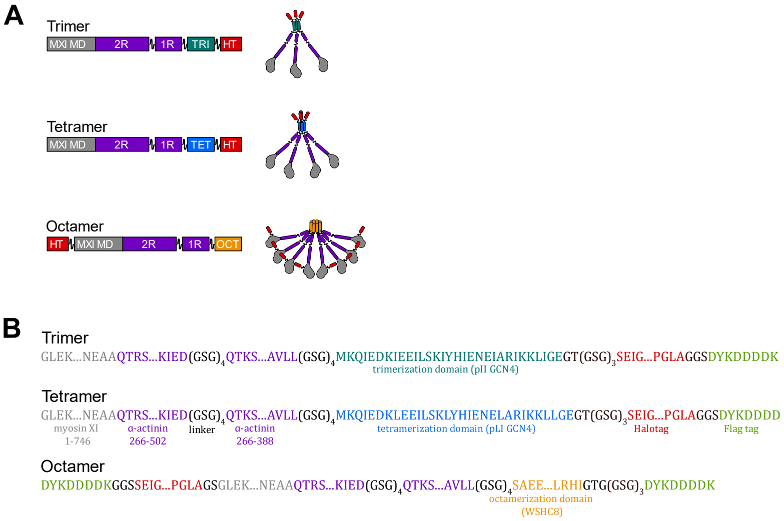

Figure S1: Details of engineered myosin constructs. (A) Block diagrams and schematics of the engineered myosin XI trimer, tetramer, and octamer. Constructs consist of a *Chara corallina* myosin XI motor domain (MXI MD), a lever arm containing two spectrin-like repeats from *Dictyostelium* α-actinin (2R), a flexible "slack" element containing one spectrin-like repeat flanked by glycine-serine-glycine linkers ($\sim$1R$\sim$), a multimerization domain (TRI, TET or OCT for the trimer, tetramer, and octamer, respectively), and a Halotag (HT). (B) Amino acid sequences of the engineered myosin constructs. Sequences are shown at the junctions between the different protein fragments within each construct.

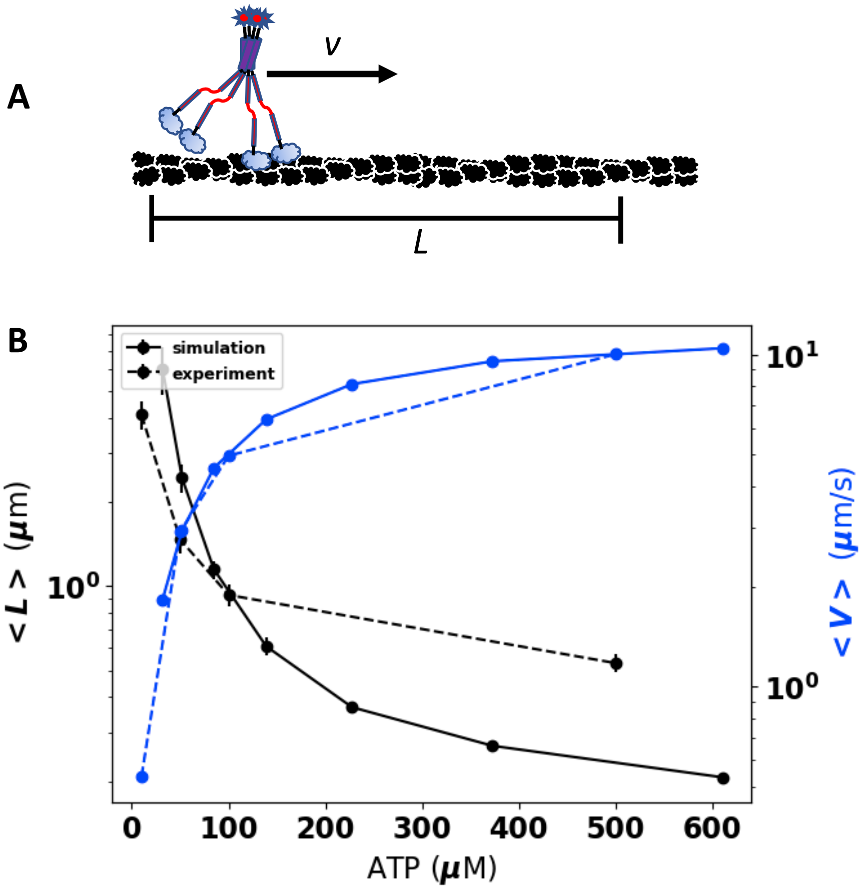

Figure S2: Simulations reproduce single-filament velocity and run length trends. (A) Schematic of single-filament measurements. Velocity (v) is the distance traveled by a motor (L) divided by the time that it stays attached. (B) Single-filament motor velocity (blue) and run length (black) from experiments (dashed lines) and simulations (solid lines) over a range of [ATP]. The final rates that we compute after 10,000 tuning steps are 1821 s^{-1}, 932 s^{-1}, 6 $s^{-1}\mu M^{-1}$ ATP.

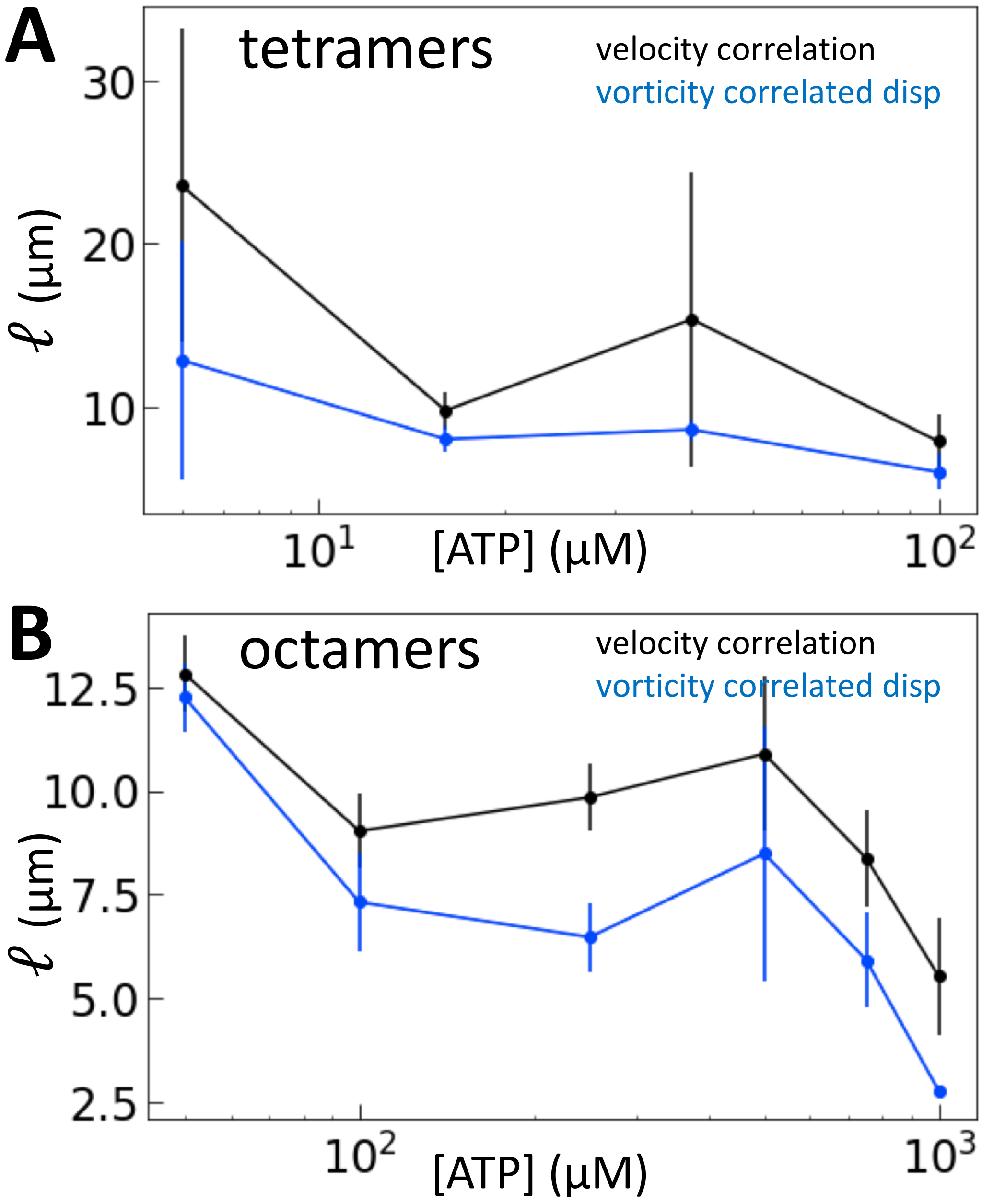

Figure S3: ℓ_{vort} **robustly captures nematic length scale.** Comparison of ℓ_{vort} as calculated in [2] and the traditional velocity correlation length at which $C_{vv} = 1/e$ for nematics driven by (A) 120 pM tetramers and (B) 50 pM octamers. Error bars are averages over five separate frames for C_{vv} and five 10 s segments for ℓ_{vort}.

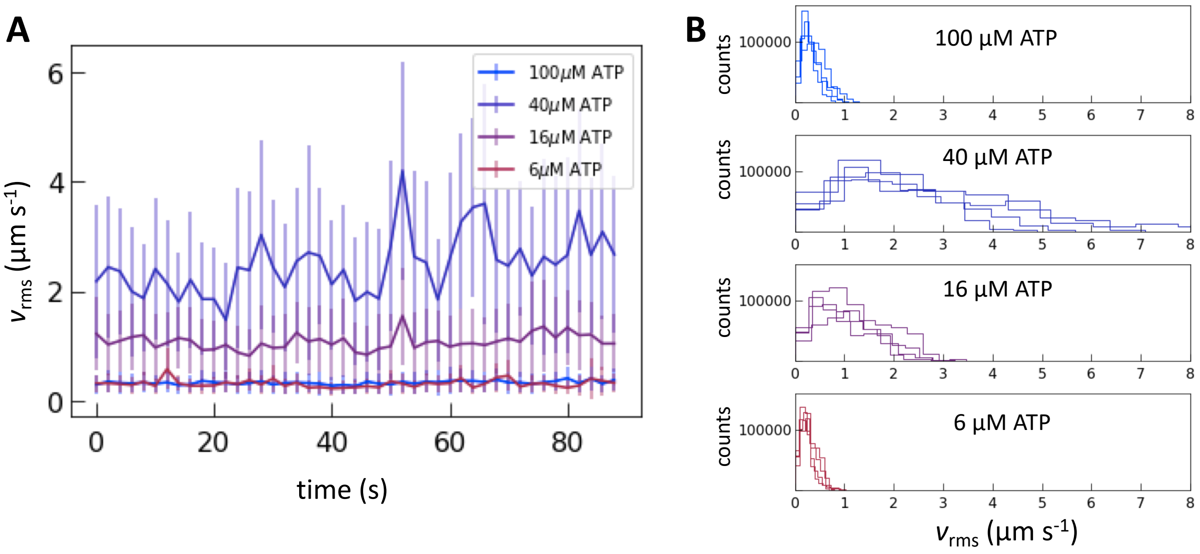

Figure S4: Nematics driven by tetrameric motors exhibit nonmonotonic speed with increasing [ATP]. (A) Average nematic speed (v_{RMS}) over time for samples driven by tetrameric motors with varying [ATP]. (B) Histograms of nematic speed for individual frames in (A) sampled at 20 s intervals.

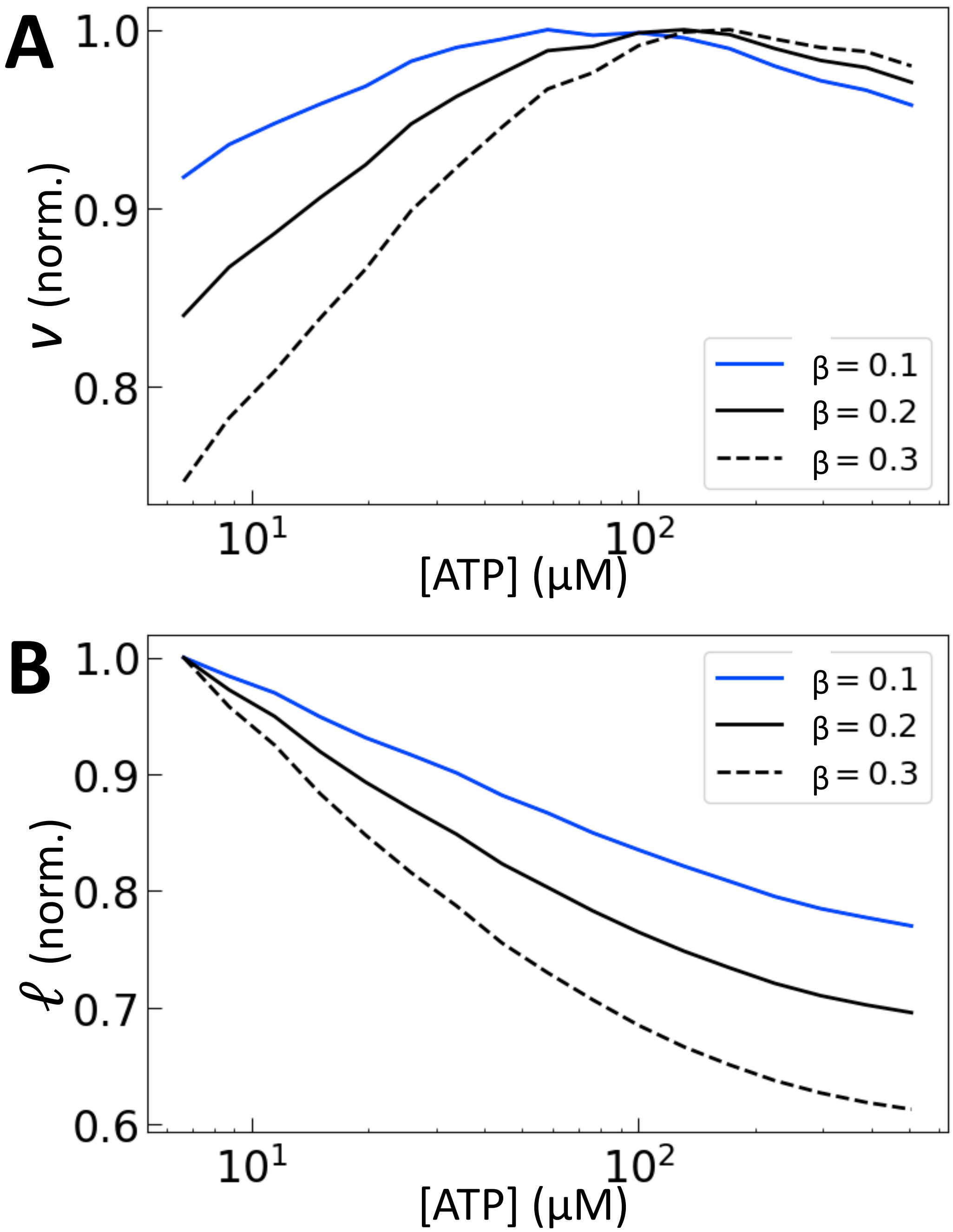

Figure S5: Dependence of (A) v **and (B)** ℓ **on** β**.**

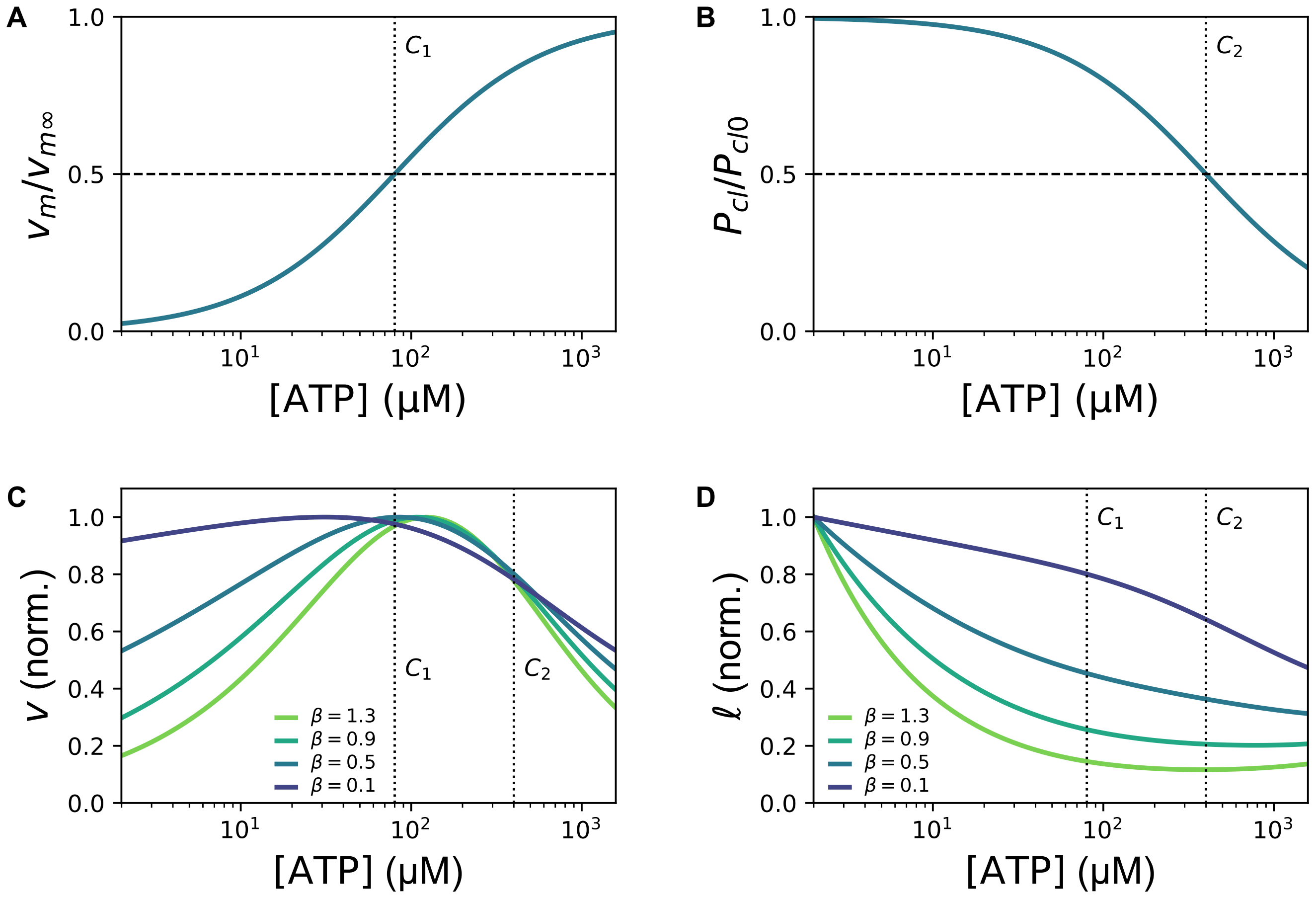

Figure S6: An alternative model for the motor velocity and crosslinking probability supports the same physical interpretation. Here, a Michaelis-Menten-like dependence on [ATP] is assumed for the (A) normalized motor velocity $v_m/v_{m\infty}$ and (B) normalized motor crosslinking probability P_{cl}/P_{cl0}. The vertical dotted lines correspond to the characteristic concentrations $C_1 = 80$ μM and $C_2 = 400$ μM at which each normalized concentration is equal to 1/2. (C) Characteristic velocity $v = \sqrt{K\alpha}/\eta$ calculated using v_m and P_{cl} according to (S5), with $v_{m\infty} = 1$, $P_{cl0} = 1$, $K_0 = 1$, $c_m = 1$, $\eta = 1$, and $\kappa = 10$. (D) Characteristic length $\ell = \sqrt{K/\alpha}$ calculated using v_m and P_{cl} according to (S4), using the same parameters.

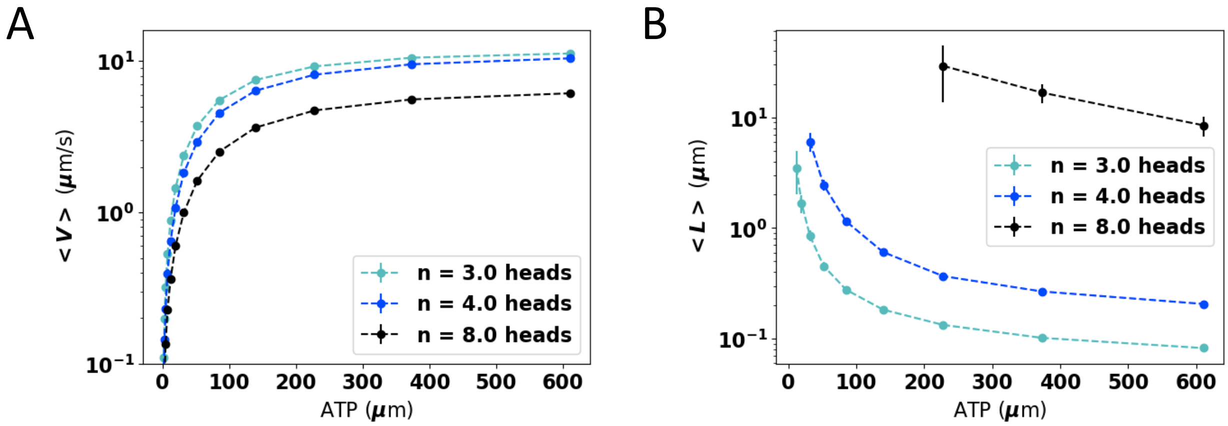

Figure S7: Motor velocity and run length depend on cluster valency. Single-filament (A) motor velocity and (B) run length measured from simulation for clusters with three, four, or eight heads per cluster.

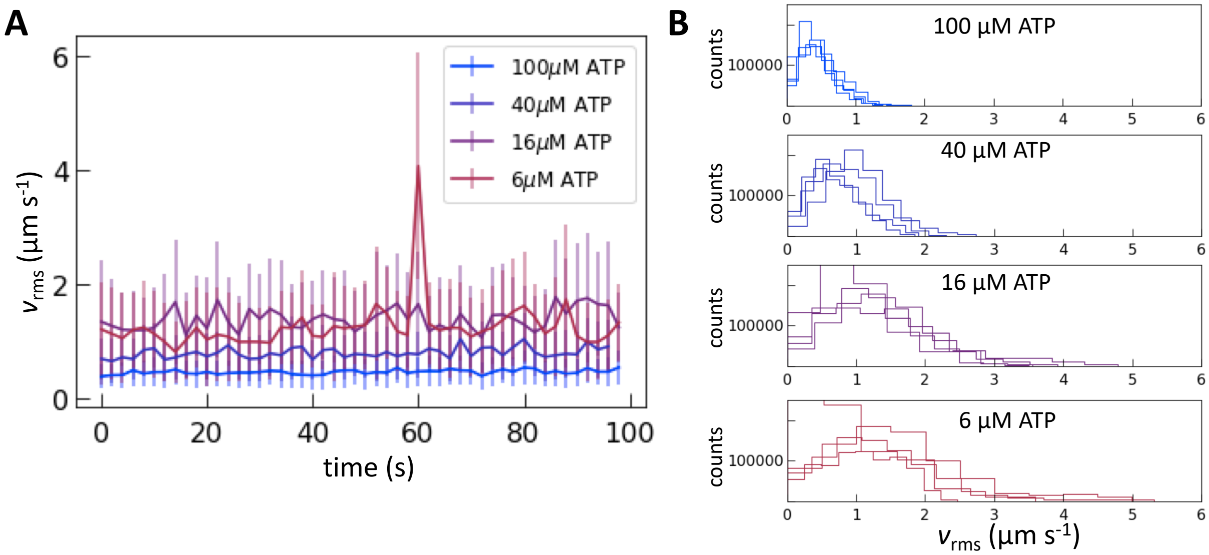

Figure S8: Nematics driven by trimeric motors exhibit nonmonotonic speed with increasing [ATP]. (A) Average nematic speed (v_{RMS}) over time for samples driven by trimeric motors with varying [ATP]. (B) Histograms of nematic speed for individual frames in (A) sampled at 2 0s intervals.

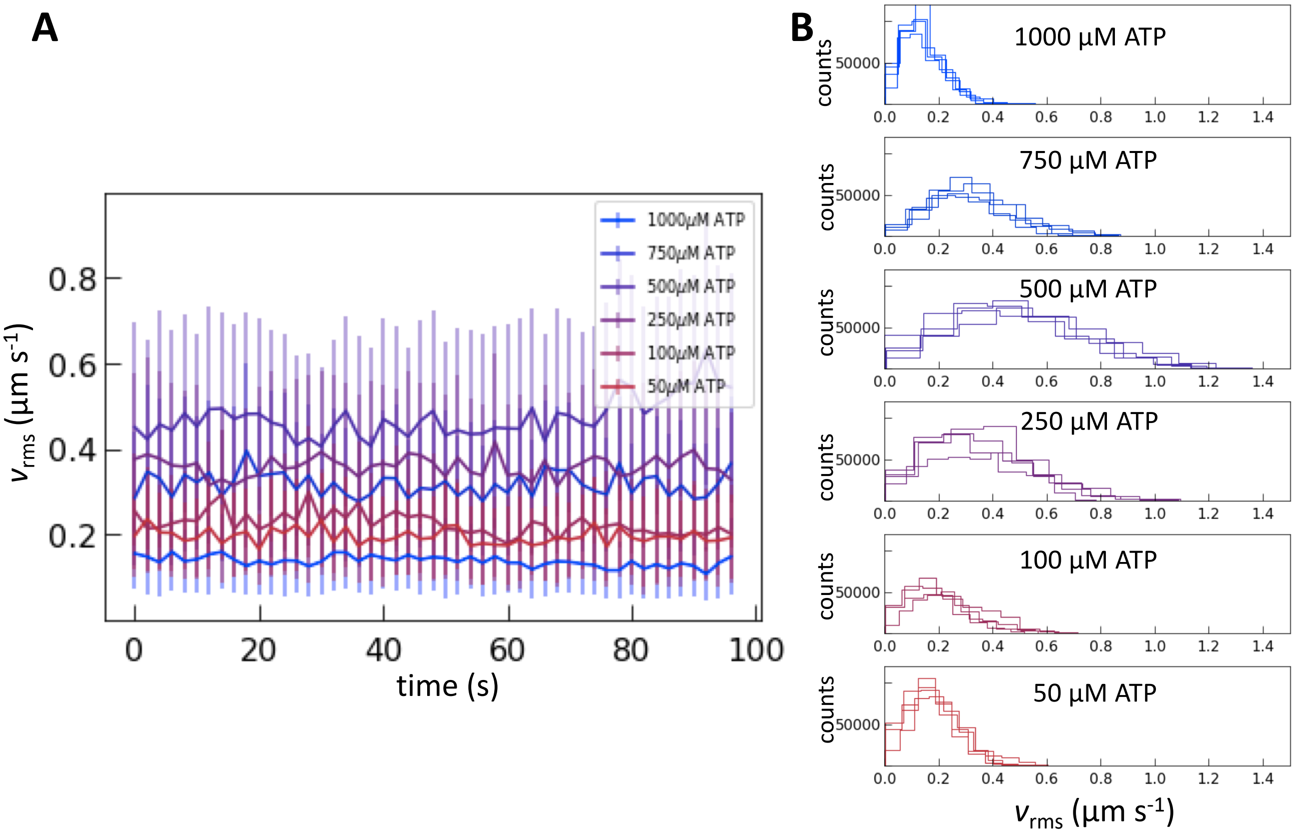

Figure S9: Nematics driven by octameric motors exhibit nonmonotonic speed with increasing [ATP]. (A) Average nematic speed (v_{RMS}) over time for samples driven by octameric motors with varying [ATP]. (B) Histograms of nematic speed for individual frames in (A) sampled at 20 s intervals.

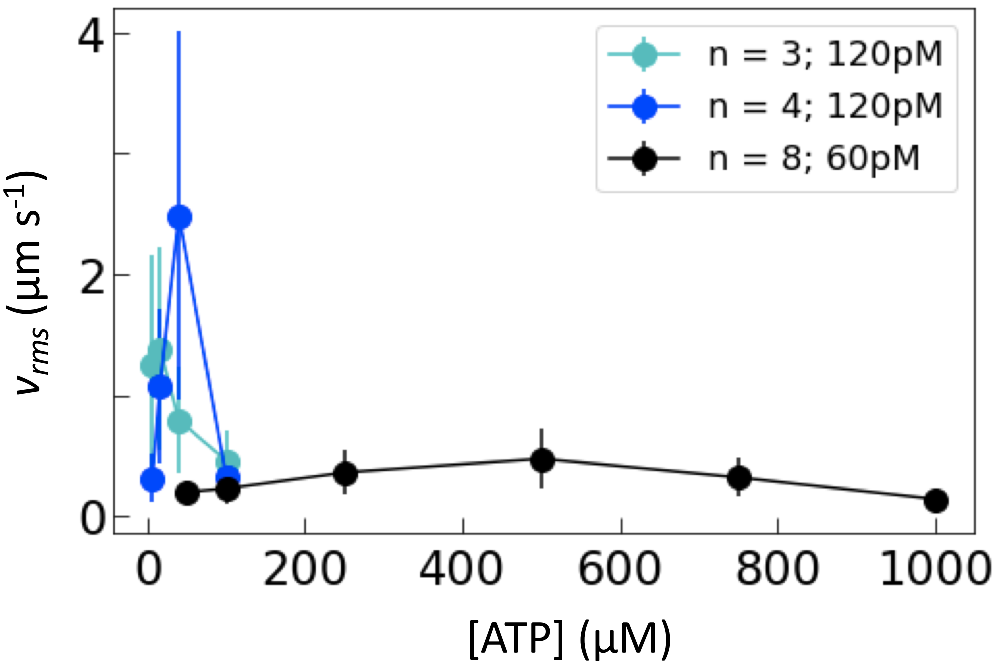

Figure S10: Results from Fig. 3E plotted on a linear [ATP] scale. v_{rms} for the experiments considered in the main text on a linear ATP scale. Error bars are standard deviations of v_{rms} over 100 s of steady-state activity.

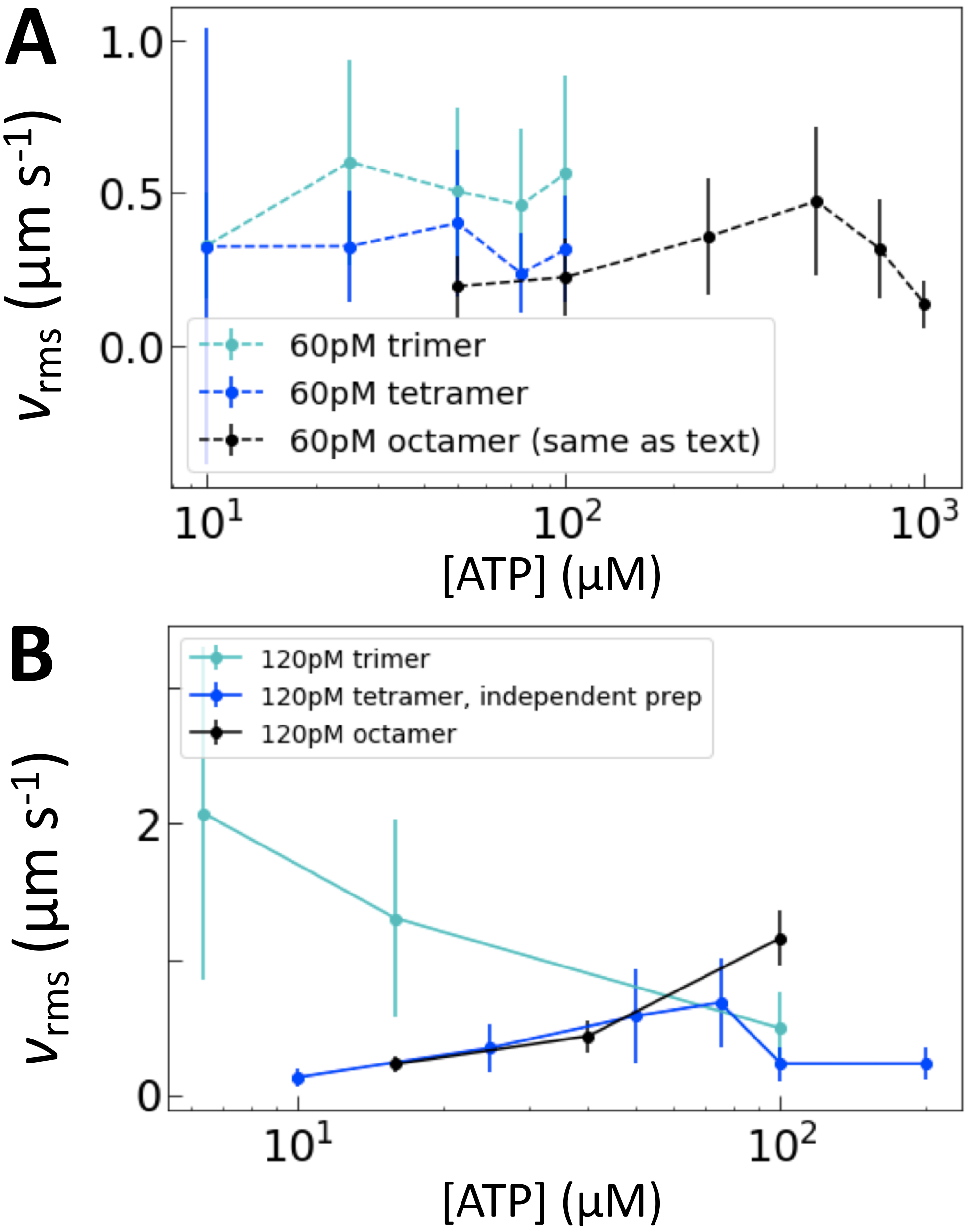

Figure S11: Replicate experiments for how motor valency tunes nematic dynamics. Measurements of v_{rms} in independent experiments with motor clusters at (A) 60 pM or (B) 120 pM for valencies as indicated. All data are different from those in the main text except for the octamers in (A), which are included as a reference. Error bars are standard deviations of v_{rms} over 100 s of steady-state activity.

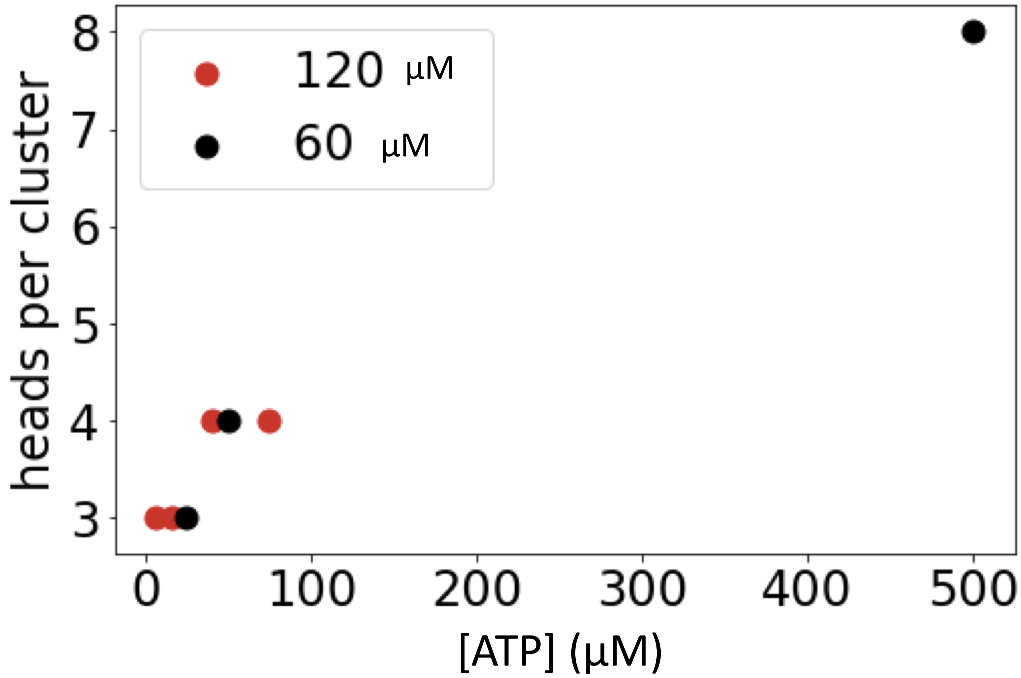

Figure S12: Peak shift is robust across replicates and motor concentrations. [ATP] at which peak speed occurred as a function of motor valency. Colors indicate cluster concentration and separate points indicate independent replicates.

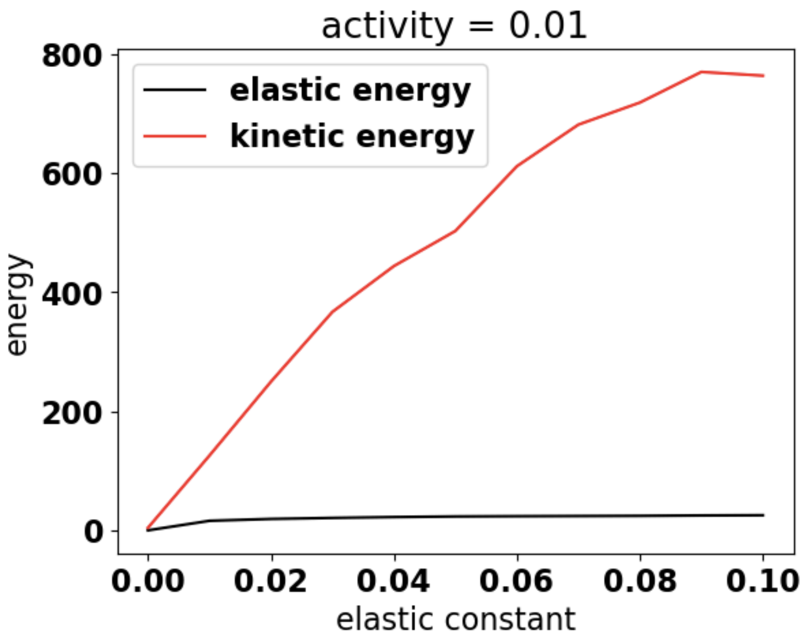

Figure S13: Nematic elasticity increases energy in the nematic. Elastic (black) and kinetic (red) energy for nematics in lattice Boltzmann simulations with constant $\alpha = 0.01$ across a range of K.
